# Clustering-Based Linear Least Square Fitting Method for Generation of Parametric Images in Dynamic FDG PET Studies

**DOI:** 10.1155/2007/65641

**Published:** 2007-10-18

**Authors:** Xinrui Huang, Yun Zhou, Shangliang Bao, Sung-Cheng Huang

**Affiliations:** ^1^The Beijing City Key Lab of Medical Physics and Engineering, Peking University, Beijing 100871, China; ^2^School of Basic Medical Sciences, Peking University, Beijing 100083, China; ^3^The Russell H. Morgan Department of Radiology and Radiological Science, School of Medicine, Johns Hopkins University Baltimore, MD 21287, USA; ^4^Department of Molecular & Medical Pharmacology, David Geffen School of Medicine, University of California, Los Angeles, CA 90095, USA

## Abstract

Parametric images generated from dynamic positron emission tomography (PET) studies are useful for presenting functional/biological information in the 3-dimensional space, but usually suffer from their high sensitivity to image noise. To improve the quality of these images, we proposed in this study a modified linear least square (LLS) fitting method named cLLS that incorporates a clustering-based spatial constraint for generation of parametric images from dynamic PET data of high noise levels. In this method, the combination of K-means and hierarchical cluster analysis was used to classify dynamic PET data. Compared with conventional LLS, cLLS can achieve high statistical reliability in the generated parametric images without incurring a high computational burden. The effectiveness of the method was demonstrated both with computer simulation and with a human brain dynamic FDG PET study. The cLLS method is expected to be useful for generation of parametric images from dynamic FDG PET study.

## 1. INTRODUCTION

Positron emission tomography (PET) is a powerful quantitative tool for in vivo imaging compounds labeled with positron emitting radioisotopes that trace biological processes in the body. However, in many occasions, the biological parameters on the image voxel level as determined by conventional statistical estimation methods suffer from large statistical uncertainty. This paper addressed this problem to make the quantitative estimation of physiological parameters more reliable.

Parameter estimation methods [1–8], such as nonlinear least square (NLS), linear least square (LLS), and graphic analysis, are used in kinetic analysis of PET data. Due to its simplicity and computational efficiency, LLS-based parameter approaches are commonly used for estimation of macroparameters such as FDG uptake rate constant $K_{i}\left(=K_{1}k_{3}/\left(K_{2}+k_{3}\right)\right)$ and distribution volume (= ($K_{1}$/$k_{2}$)(1 + $k_{3}$/$k_{4}$)) for reversible ligand-receptor PET study. Regardless of whether the data is from ROI or from a single voxel, the noise-induced bias has been reported in previous studies [9–11]. The application of conventional LLS method for generation of microparameter images of FDG kinetic model could be limited by high noise levels of pixel kinetics. The option of reducing the noise by increasing the injection dose is limited by clinical practice. Averaging over a larger volume or setting a big voxel size could reduce the noise, but it is limited by tissue heterogeneity and the partial volume effect. The clustering-based analyses developed recently [12–17] reduced the noise effectively because these methods averaged the data over a large volume that included many tissues with similar tracer kinetics or physiological characteristics. The clustering operation automatically segments the tissues into different clusters, within each the time activity curves (TACs) of all voxels have a similar shape. The combination of clustering analysis and the LLS method may give the required robustness and reliability in the parameter estimation and yet without significantly increasing the computation burden for generation of parametric images.

Comparing with previous methods, clustering analysis for kinetics (CAKS) method was originally developed based on the principle component analysis (PCA) [12, 13] with only two principle components. A variation of the original CAKS method was based on the mixed-Gaussian model [14], and was applied at the voxel level. Nonlinear ridge regression with spatial constraint (NLRRSC) was used for nonlinear least square (NLS) estimation after hierarchical cluster analysis [15]. Simultaneous estimation (SIME) or simultaneous estimation with postestimation (SIMEP) method was a simultaneous estimation approach using a K-means-like cluster analysis [16]. In the present study, a combination of K-means and hierarchical cluster analysis is used for clustering dynamic FDG PET data, and the kinetics of clusters of high signal-to-noise ratio are applied to regression matrix for LLS to produce parametric images. The method was verified using computer simulated data and a human FDG PET data set to show its superior performance compared to the conventional LLS or Patlak graphic analysis.

## 2. METHOD

### 2.1. Modeling theory

The following three-compartment FDG model [18] has been shown before to be appropriate for analysis of dynamic FDG PET data [1,19,20],

(1) $$\begin{array}{l} \frac{dC_{e}}{dt}=K_{1}C_{p}-(k_{2}+k_{3})C_{e},\\ \frac{dC_{m}}{dt}=k_{3}C_{e},\\ C_{t}=C_{e}+C_{m},\\ C_{\text{p}\text{e}\text{t}}=(1-V_{B})C_{t}+V_{B}C_{p}, \end{array}$$

where $C_{e}$, $C_{m}$, and $C_{p}$ are, respectively, the concentrations of free FDG, FDG-6-phosphate in tissue, and the FDG concentration in plasma. $C_{t}$ is the total radioactivity in tissue, $C_{\text{p}\text{e}\text{t}}$ is total counts from the whole field of view (FOV). $K_{1}$ is a transfer constant for free FDG from plasma into tissue, $k_{2}$ is a rate constant for free FDG from tissue back to plasma, and $k_{3}$ is rate constant for FDG phosphorylated into FDG-6-PO_4_.$V_{B}$ is fractional volume of blood in tissue $\left(0\leq V_{B}\leq 1\right)$.

Then, the cerebral glucose metabolic rate can be calculated as

(2) $$\text{M}\text{R}\text{G}\text{l}c=\frac{K_{1}k_{3}}{k_{2}+k_{3}}\cdot \frac{\left[\text{G}\text{l}c\right]}{\text{L}\text{C}}=K_{i}\cdot \frac{\left[\text{G}\text{l}c\right]}{\text{L}\text{C}},$$

where [Glc] is the glucose concentration in plasma. The lumped constant (LC) is usually a constant. Therefore, to get glucose metabolic rate, one only needs to calculate the uptake rate constant $K_{i}$.

The linear description of (1) can be written as follows:

(3) $$\begin{array}{ll} C_{t}\left(t\right) & =K_{1}\int_{0}^{t}C_{p}\left(\tau \right)d\tau +K_{1}k_{3}\iint_{0}^{t}C_{p}\left(\theta \right)d\theta d\tau \\ & \;-\left(k_{2}+k_{3}\right)\int_{0}^{t}C_{t}\left(\tau \right)d\tau +\varepsilon ,\\ C_{t}\left(t\right) & =\;\;\frac{C_{pet}\left(t\right)-V_{B}C_{p}}{1-V_{B}}. \end{array}$$

cLLS is a modified linear least square (LLS) fitting method that incorporates a clustering-based spatial constraint for generation of parametric images from dynamic PET data of high noise levels. The 3D autoclustering processing was applied to the smoothed dynamic PET data by a method that combines K-means and hierarchical clustering with average linkage. The number of clusters was determined by referring to previous work [15]. The average TAC and $V_{B}$ of each cluster ($C_{\text{p}\text{e}\text{t}\_\text{c}\text{l}\text{u}\text{s}\text{t}\text{e}\text{r}}$ and $V_{B\_\text{c}\text{l}\text{u}\text{s}\text{t}\text{e}\text{r}}$) were then determined and were used to improve the LLS method as shown below in (4) and (6) (note that all the tissue TACs in the following equations are the measurements after being corrected for $V_{B\_\text{c}\text{l}\text{u}\text{s}\text{t}\text{e}\text{r}}$ (e.g., $C_{t}\left(t\right)=(C_{\text{p}\text{e}\text{t}}\left(t\right)-V_{B\_\text{c}\text{l}\text{u}\text{s}\text{t}\text{e}\text{r}}C_{p})/\;(1-V_{B\_\text{c}\text{l}\text{u}\text{s}\text{t}\text{e}\text{r}})$, $C_{t\_\text{c}\text{l}\text{u}\text{s}\text{t}\text{e}\text{r}}\left(t\right)=(C_{\text{p}\text{e}\text{t}\_\text{c}\text{l}\text{u}\text{s}\text{t}\text{e}\text{r}}\left(t\right)-V_{B\_\text{c}\text{l}\text{u}\text{s}\text{t}\text{e}\text{r}}C_{p})/\left(1-V_{B\_\text{c}\text{l}\text{u}\text{s}\text{t}\text{e}\text{r}}\right)$),

(4) $$\begin{array}{ll} C_{t}\left(t\right)= & K_{1}\int_{0}^{t}C_{p}\left(\tau \right)d\tau +K_{1}k_{3}\int_{0}^{t}\int_{0}^{\tau }C_{p}\left(\theta \right)d\theta d\tau \\ & -\left(k_{2}+k_{3}\right)\int_{0}^{t}C_{t\_\text{c}\text{l}\text{u}\text{s}\text{t}\text{e}\text{r}}\left(\tau \right)d\tau +\varepsilon . \end{array}$$

The conventional way of calculating the value of $K_{i}$ as $K_{1}k_{3}/\left(k_{2}+k_{3}\right)$ may have a large error propagation, because the estimates of $k_{2}$ and $k_{3}$ determined from high noise TAC usually have large variability. In order to obtain a more robust $K_{i}$ estimate, (3) can be rearranged for estimating $K_{i}$ directly as

(5) $$\begin{array}{ll} \int_{0}^{t}C_{t}\left(\tau \right)d\tau = & \frac{K_{1}}{k_{2}+k_{3}}\int_{0}^{t}C_{p}\left(\tau \right)d\tau +K_{i}\int_{0}^{t}\int_{0}^{\tau }C_{p}\left(\theta \right)d\theta d\tau \\ & -\frac{1}{k_{2}+k_{3}}C_{t}\left(t\right)+\varepsilon . \end{array}$$

Substituting $C_{t}$ with $C_{t\_\text{c}\text{l}\text{u}\text{s}\text{t}\text{e}\text{r}}$ (of lower noise level) on the right side of (5) is expected to improve further the estimate of $K_{i}$,

(6) $$\begin{array}{ll} \int_{0}^{t}C_{t}\left(\tau \right)d\tau = & \frac{K_{1}}{k_{2}+k_{3}}\int_{0}^{t}C_{p}\left(\tau \right)d\tau +K_{i}\int_{0}^{t}\int_{0}^{\tau }C_{p}\left(\theta \right)d\theta d\tau \;\\ & -\frac{1}{k_{2}+k_{3}}C_{t\_\text{c}\text{l}\text{u}\text{s}\text{t}\text{e}\text{r}}+\varepsilon . \end{array}$$

### 2.2. Validation method

**(1) Computer simulation**

Time activity curves for 100 pixels within a cluster were simulated to investigate the influence of noise on parameter estimation with conventional LLS and cLLS at various noise levels. The regular FDG model was used for the simulation.

Parameters used were $K_{1}$ = 0.13 (mL/min/g), $k_{2}$ = 0.08 (1/min), $k_{3}$ = 0.05 (1/min). The dynamic PET scan time sequence (4×0.5 min, 4×2 min, 10×5 min) was the same as the one commonly used in human FDG study. A plasma TAC from a real human FDG study was used as the input function. The noise-free tissue TAC was generated according to the analytical solution of the model, that is,

(7) $$C_{t}\left(t\right)=\frac{K_{1}}{k_{2}+k_{3}}\left(k_{3}+k_{2}e^{-\left(k_{2}+k_{3}\right)}\right)⊗C_{p}\left(t\right).$$

Pseudorandom Gaussian noise was added to the TAC according to the noise variance formula shown by Chen et al. [21] and Feng et al. [22] before. That is, the variance of the noise was proportional to the radioactivity concentration and inversely proportional to the scan duration ($\Delta t_{i}$),

(8) $$\sigma^{2}\left(t_{i}\right)=\frac{\alpha \times C_{t}\;\;(t_{i})\;e^{0.693t_{i}/\lambda }}{\Delta t_{i}},$$

where $\sigma^{2}\left(t_{i}\right)$ is the variance of ith scan at its midtime ($t_{i}$), α is the proportionality constant that determines the overall noise level in a TAC, and *λ* is the physical half-life of FDG (= 110 min).

In this simulation, α was set as 0.1, 0.2, 1.0, and 1.5 to yield noisy TACs at various noise levels. The simulated TACs were then processed using LLS and cLLS, separately to give the estimates of the two methods. For the cLLS, the cluster average tissue TAC in (4) and (6) used was the average TAC of the 100 simulated TACs. The estimated parameters for each of the 100 pixels were obtained for each noise level. Bias and root mean squared error (RMSE) were used as criteria to evaluate the reliability of cLLS. The percentages of RMSE and bias are defined as [15]

(9) $$\begin{array}{l} \text{RMSE}\%=\frac{1}{p}\sqrt{\frac{\sum_{i=1}^{N}{\left(p_{i}-p\right)}^{2}}{N-1}}\times 100\%,\\ \text{Bias}\%=\frac{1}{p}\sum_{i=1}^{N}\frac{\left(p_{i}-p\right)}{N}\times 100\%, \end{array}$$

where $p_{i}$ is the parameter estimation result of the ith simulated pixel TAC at one noise level, p is the “true” parameter value for the simulation, and N is the number of pixels in the cluster (i.e., N = 100).

**(2) Clinical data validation**

A set of dynamic FDG PET data was acquired from a normal volunteer with an ECAT EXACT HR+ PET scanner (axial field of view = 15.5 cm; intrinsic full-width-at-half-maximum (FWHM) at the center = 4.3 mm) in 3D acquisition mode. Before FDG administration, transmission scanning was performed using ^68^Ge line sources for attenuation correction. Dynamic emission scans (4×0.5 min, 4×2 min, 10×5 min) were initiated simultaneously with an IV injection of 155 MBq FDG. For each PET scan frame, 63 transaxial images (128×128 pixel; pixel size 1.471 mm; 2.425-mm plane thickness) were reconstructed using a filtered back-projection algorithm with a Hanning filter (cut-off frequency of 0.3 cycle per projection element), resulting in an in-plane spatial resolution of∼8 mm FWHM. Dead time, scatter, and measured attenuation corrections were applied. Arterial blood samples were collected via a catheter in the radial artery during the study.

The acquired image data was processed with the following procedure.

(1) After the mean images were obtained by averaging all the frames of the time series, they were smoothed with a 6-mm FWHM Gaussian filter to get an image mask of the brain using SPM99. All PET dynamic images were masked to zero out all the pixels outside of the head.

(2) The clustering of the 3D PET dynamic data follows the procedure described as follows. (a) 3D smoothing of the masked PET 3D data of each frame; (b) classifying pixel TACs of the masked and smoothed PET 3D dynamic data into 15 clusters with the K-means method; (c) classifying the 15 average TACs from the 15 clusters into 4 final clusters (white matter, gray matter, scalp, and vasculature) with the average linkage hierarchical clustering method.

(3) The cluster average TAC was obtained as the average of all the voxel TACs in each cluster. Fitting the cluster average TAC with the FDG model (1) using the Levenberg-Marquardt algorithm gave the estimates of the parameters, $K_{1}$, $k_{2}$, $k_{3}$, $V_{B}$, for each cluster.

(4) After correcting the dynamic PET data and the clustered average TAC of each cluster with $V_{B\_\text{cluster}}$, the parametric images ($K_{1}$, $k_{2}$, and $k_{3}$) were estimated using (3) and (4). $K_{i}$ parametric image was estimated from $K_{1}k_{3}/(k_{2}+k_{3})$ for the conventional LLS and according to (6) for cLLS.

(5) To validate the robustness of the cLLS method, the parameters estimated by cLLS were compared against those from the conventional LLS method and those from the Patlak graphical method.

## 3. RESULTS

**(1) Results of the clustering**

**Figure 1:**
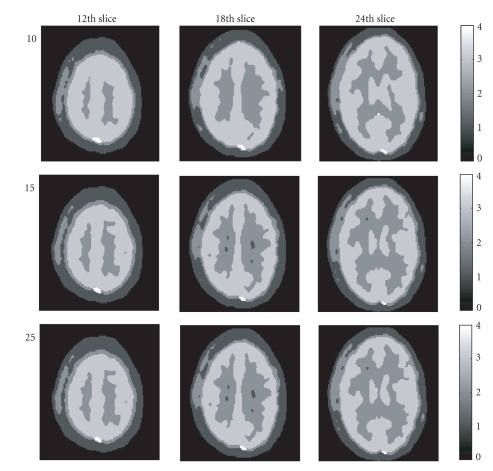
The 12th slice, 18th slice, and 24th slice of 3D PET dynamic data clustered image.

After the clustering processing, a brain image is classified into 4 clusters. Figure 1 shows the cluster image results at the 12th, 18th, and 24th slices. To investigate how the K-means' results effect the average linkage output, various numbers of clusters (10, 15, and 25) from the K-means clustering were tested and the results from the average linkage were shown in different rows in Figure 1. Some detail anatomic features were lost with 10 clusters. Although some voxels were assigned to the wrong cluster with the use of 25 clusters, the differences cannot be exactly distinguished between 15 clusters and 25 clusters. To get an idea of how well separated the resulting clusters are, we can make a silhouette plot using the final cluster indices output. The silhouette plot displays a measure of how close each point in one cluster is to points in the neighboring clusters. A quantitative way to compare the different solutions is to look at the average silhouette values for the different cases. The bigger average silhouette value indicates the better cluster result, for example, the average silhouette values of 12th slice are 0.8084, 0.8132, and 0.8177, respectively, for the final cluster results from 10, 15, and 25 clusters from the K-means clustering.

Considering the computation efficiency and simplicity simultaneously, the estimated parameters of each cluster, from the average linkage results with 15-cluster K-means results, are summarized in Table 1.

**(2) Results of the simulation**

**Figure 2:**
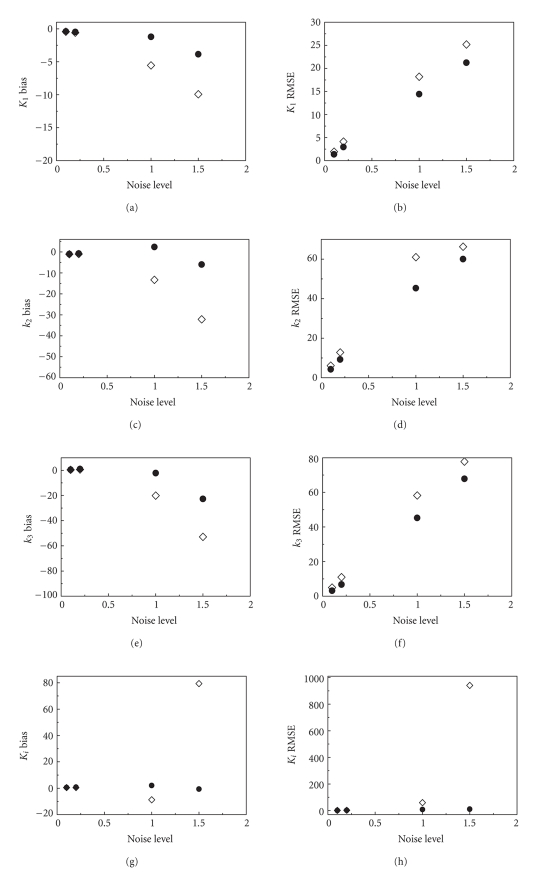
Bias and RMSE of estimated parameters for the simulation data at different noise levels (diamond for LLS results; filled circle for cLLS results).

The estimated parameters (by LLS and by cLLS) from the simulated data at different noise levels are listed in Table 2. The bias and RMSE of the estimates are shown in Figure 2. The absolute values of percent bias and percent RMSE of the estimates are small for both methods when the TAC noise level is low. For higher noise levels, the results from the conventional LLS quickly deteriorate, while the estimated uptake rate constant $k_{i}$ from cLLS remains stable (i.e., the absolute values of bias and RMSE with the cLLS are both small).

**(3) Results of clinical data validation**

**Figure 3:**
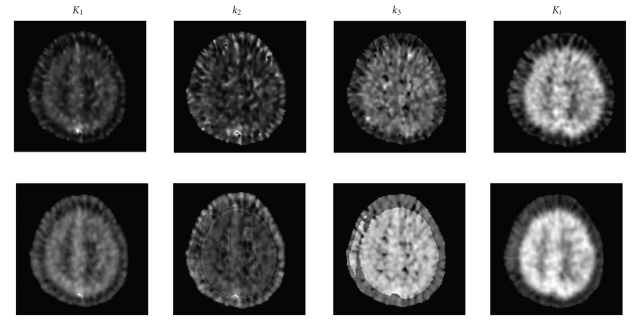
The parametric images of the 12th slice in clinical 3D FDG PET dynamic data, the first row is the images from LLS and the second row is the images from cLLS.

The parametric images of the 12th slice as estimated by LLS and by cLLS are shown in Figure 3. It is evident that the parametric images from cLLS have a better image quality and higher SNR.

To quantitatively compare the parametric images, we applied a number of small ROIs to the parametric images, and compared the mean and standard deviation of the pixel values within the ROIs. The results are shown in Table 3. Compared to LLS, cLLS gives higher means and lower SDs for the estimates.

To investigate noise effects on real data, big-to-small ROIs were selected and kinetically analyzed. The ROI parameter values were obtained with three methods separately: conventional nonlinear regression method fitting the average TAC of all ROI pixel TACs; LLS method fitting each ROI pixel TAC and giving out the mean of all pixel parameters; cLLS method fitting each ROI pixel TAC with the average ROI TAC substituting the cluster average tissue TAC in (4) and (6). RMSE of estimates ($K_{1}$, $k_{2}$, $k_{3}$, and $K_{i}$), with conventional nonlinear regression method as reference, the RMSE of cLLS estimate is (22.7% for $K_{1}$; 12.6% for $k_{2}$; 16.5% for $k_{3}$, and 91.2% for $K_{i}$) than that of LLS (paired t-test, P<.01).

**(4) Correlation comparison of different methods**

**Figure 4:**
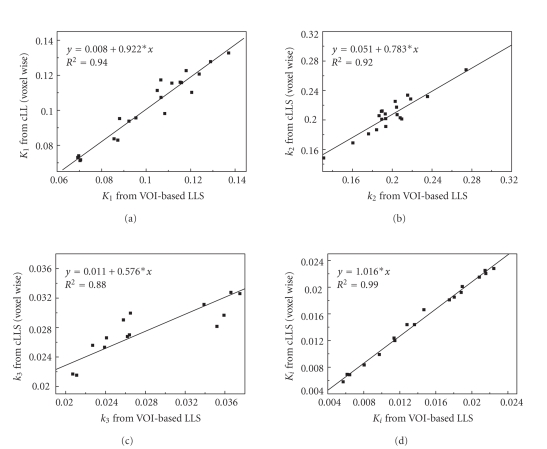
The correlation of voxel-based average parameters of VOIs in parametric image space with the parameters derived from VOI kinetic analysis with conventional LLS fitting.

**Figure 5:**
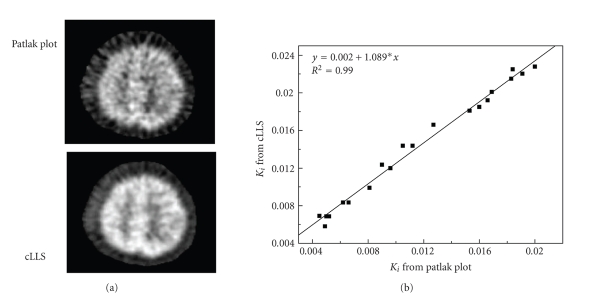
The correlation of the parameter comparison between cLLS and Patlak methods in parametric image space.

Squared correlation coefficients ($R^{2}$) between ROI averages of parametric images (by cLLS) and parameters estimated by ROI kinetic analysis (with LLS) are 0.94 for $K_{1}$, 0.92 for $k_{2}$, 0.88 for $k_{3}$, and 0.99 for $K_{i}$ (Figure 4). The $K_{i}$ estimate for each pixel from cLLS also correlates well with that from the Patlak graphical analysis ($R^{2}$ = 0.99, y = 0.002+1.089*x) (Figure 5).

## 4. DISCUSSION

**(1) Advantage of the clustering processing in the cLLS method**

From the result shown in Figure 1, one sees that the clustering technique in our cLLS method clearly segmented the dynamic PET images into 4 clusters. Compared to previous works based on subjective ROI drawing that is labor intensive and error prone, our clustering technique performed well. In addition, the process of clustering is not subject to tissue heterogeneity, with which manual ROI method is difficult to deal. Comparing with the relevant studies that the clustering method has been used in dynamic PET FDG data analysis before, the novelty of our study is the integration of modified clustering method and LLS based on voxel level.

**(2) High accuracy and reliability of cLLS**

From the simulated results in Figure 2, one can see that the absolute values of percent bias and percent RMSE of the estimates from cLLS are usually smaller than those of LLS at all noise levels. The use of the cluster average TAC on the right-hand-sides of (4) and (6) is considered to be the main factor that accounts for the improvement. This result is in agreement with those previously reported by Kimura et al. [12] which showed that direct parameter estimation using the conventional LLS would result in a large bias compared with the linearization method with cluster analysis.

**(3) cLLS analysis saving more calculation time**

For cLLS, the right-hand-sides of (4) and (6) are calculated only 4 times (the number of clusters) for each study, while the same computation (3) needs to be repeated for each voxel for LLS. The calculated time of cLLS is about 2 hours, less than about 6 hours of LLS, when dealing with the clinical data. The program was edited in Matlab 6.5 language under Windows XP system and run on the PC with 2.0 G RAM and P4 CPU.

In conclusion, the present study showed that the cLLS method is of high computational efficiency and provides estimates of high statistical reliability. The cLLS method is thus expected to be useful for generation of parametric images from dynamic brain FDG PET of high noise levels. In the paper, the data of a normal volunteer was used to validate the effectiveness of the method, because the anatomic structure of a normal volunteer is clear for us especially when we understand the clustering result. In future, we also can try some dynamic PET FDG data of patients with brain disorder to validate the cLLS method.

## Figures and Tables

**Table 1:** The parameters of every cluster with NLS fitting.

	Cluster 1	Cluster 2	Cluster 3	Cluster 4
$K_{1}$	0.088	0.109	0.170	0.222
$k_{2}$	0.343	0.249	0.284	0.629
$k_{3}$	0.023	0.042	0.048	0.013
$V_{2}$	0.042	0.052	0.070	0.137
$K_{i}$	0.006	0.016	0.025	0.004

**Table 2:** Mean of estimated parameters for the simulation data at different noise levels (“true” parameter values: $K_{1}$ = 0.130, $k_{2}$ = 0.080, $k_{3}$ = 0.050, $K_{i}$ = 0.050).

	$K_{1}$		$k_{2}$		$k_{3}$		$K_{i}$	
	LLS	cLLS	LLS	cLLS	LLS	cLLS	LLS	cLLS
0.1	0.129	0.130	0.079	0.079	0.050	0.050	0.050	0.050
0.2	0.129	0.129	0.079	0.079	0.050	0.051	0.050	0.050
1	0.123	0.128	0.069	0.082	0.034	0.049	0.046	0.051
1.5	0.117	0.124	0.054	0.074	0.024	0.038	0.088	0.050

**Table 3:** Pixelwise comparison on mean and SD of parametric images (paired t-test, P<.05).

		Mean	SD	SD
				lower
$K_{1}$	cLLS	0.111	0.024	18.6%
	LLS	0.104	0.029	
$k_{2}$	cLLS	0.150	0.051	14.5%
	LLS	0.123	0.059	
$k_{3}$	cLLS	0.031	0.008	33.6%
	LLS	0.029	0.013	
$K_{i}$	cLLS	0.023	0.004	89.7%
	LLS	0.020	0.035	
